# *In Vitro* Evaluation of the Potential Use of Propolis as a Multitarget Therapeutic Product: Physicochemical Properties, Chemical Composition, and Immunomodulatory, Antibacterial, and Anticancer Properties

**DOI:** 10.1155/2019/4836378

**Published:** 2019-12-12

**Authors:** Soumaya Touzani, Walaa Embaslat, Hamada Imtara, Abdalsalam Kmail, Sleman Kadan, Hilal Zaid, Ilham ElArabi, Lyoussi Badiaa, Bashar Saad

**Affiliations:** ^1^Physiology-Pharmacology, University of Fez, P.O. Box 1796 Fez Atlas, Fez, Morocco; ^2^Faculty of Allied Medical Sciences, Arab American University Palestine, P.O. Box 240, Jenin, State of Palestine; ^3^Qasemi Research Center- Al-Qasemi Academic College and Faculty of Arts and Sciences, Arab American University Palestine, P.O. Box 240, Jenin, State of Palestine

## Abstract

Propolis is a resin that honeybees produce by mixing saliva and beeswax with exudate gathered from botanical sources. The present *in vitro* study investigated the potential use of propolis as a multitarget therapeutic product and the physicochemical properties, chemical composition, and immunomodulatory, antioxidant, antibacterial, and anticancer properties of a propolis extract from the northern Morocco region (PNM). Pinocembrin, chrysin, and quercetin were the main phenolic compounds of PNM as measured in HPLC. The PNM showed significant inhibitory effects against all tested Gram-positive and Gram-negative strains and showed high antioxidant activities by scavenging free radicals with IC50 (DPPH = 0.02, ABTS = 0.04, and FRAP = 0.04 mg/ml). In addition, PNM induced a dose-dependent cytostatic effect in MCF-7, HCT, and THP-1 cell lines at noncytotoxic concentrations with IC50 values of 479.22, 108.88, and 50.54 *μ*g/ml, respectively. The production of tumor necrosis factor-*α* (TNF-*α*) and interleukin-6 (IL-6) was decreased in a dose-dependent manner in LPS-stimulated human peripheral blood mononuclear cells (PBMNCs), whereas the production of the anti-inflammatory interleukin-10 (IL-10) was increased in a dose-dependent manner reaching 15-fold compared to the levels measured in untreated PBMNCs. Overall, the results showed that the traditionally known multitarget therapeutic properties of the PNM seem to be mediated, at least in part, through cytostatic, antibacterial, and immunomodulatory effects.

## 1. Introduction

For centuries, bee products have been used in all traditional medical systems. Honey and propolis (also known as bee glue) have been the subject of extensive studies for their health-promoting properties. Propolis is a resinous substance collected by bees from their surrounding plants [1, 2]. Hence, it contains a variety of phytochemicals that are produced by plants through primary and secondary pathways. Bees use propolis as an insulating material and as a sealant for unwanted open spaces in the hive. Due to its high antimicrobial properties, it plays an essential role in the hive defense [2]. In recent years, propolis has attracted much attention as a valuable or potential substance used in medicine and cosmetic products. It is known to exhibit valuable therapeutic and biological activities. These include, but not limited to, anticancer, antitumor, antioxidant, antimicrobial, antiulcer, and antifungal properties. It was also reported to have hepatoprotective, cardioprotective, antihypertensive, hypoglycemic, and hypolipidemic activities [3, 4].

The chemical composition of propolis is quite complex. More than 300 phytochemicals have been identified so far in propolis. These include polyphenols, phenolic aldehydes, sesquiterpene quinines, coumarins, amino acids, steroids, and inorganic compounds. The chemical composition of propolis depends on the collecting time, location, and plant source. As a result, biological activities of propolis gathered at various times and from different phytogeographical areas vary greatly [3–5]. For example, propolis samples collected from temperate areas contain flavonoids pinocembrin, chrysin, ferulic acid, cinnamic acid, and caffeic acid [6, 7], whereas propolis from tropical regions is rich in prenylated derivatives of benzophenones, *p*-coumaric acid, lignans, and diterpenes [8]. Many of the propolis active components exhibited *in vitro* and *in vivo* anti-inflammatory effects through affecting common and/or distinct anti-inflammatory pathways. One example is the pathway that involves the Toll-like receptors (TLRs), which recognize various microbial receptors called pathogen-associated molecular patterns (PAMPs). Consequently, proinflammatory cytokines are released through the activation of NF-kB and other transcriptional factors [9]. This response is mediated by B and T cells and results in pathogen-specific adaptive immunity [10]. Propolis-derived neovestitol, an isoflavonoid, inhibited nitric oxide (NO) production and reduced proinflammatory cytokine levels from lipopolysaccharide- (LPS-) stimulated macrophage cell line RAW264.7 [11]. Propolis-derived caffeic acid, phenethyl ester, quercetin, and hesperidin strongly reduced DNA synthesis and the production of IL-1, IL-12, IL-2, and IL-4 and enhance the production of transforming growth factor-*β* (TGF-*β*) from T cells [12]. In addition, apigenin, galangin, and pinocembrin, isolated from propolis collected in southern Brazil [13], were found to modulate the production of proinflammatory *in vitro*. Apigenin decreased the mRNA levels of IL-1, IL-6, and TNF-*α* in human THP-1-derived macrophages [14]. The levels of these cytokines were also significantly reduced by pinocembrin in macrophage cell line RAW264.7, whereas IL-10 was significantly increased [15]. In the same RAW264.7 line, the level of IL-6 and TNF-*γ* cytokines was clearly inhibited by galangin [16]. *In vivo,* propolis administration to C57BL/6 mice for 14 days led to reduced production of IL-1, IL-6, IL-2, IL-10, and IFN-*γ* by spleen cells [17]. In addition, ethanolic extract of Brazilian propolis reduced the expression of IL-17 in collagen-induced arthritis in mice [18]. Thus, bee propolis and its constituents can be considered as potential natural anti-inflammatory agents that act by modulating immune responses.

Besides the anti-inflammatory effects, propolis-derived flavonoids exhibit powerful antioxidant activities and are capable of scavenging free radicals and thereby protecting the cell membrane against lipid peroxidation. In addition, propolis extracts were found to protect the liver in rats against carbon tetrachloride (CCl) injury. It seems that propolis exerts these hepatoprotective effect through the inhibition of phase I enzymes and the induction of phase II enzymes [19].

Many studies have been conducted on the propolis of Morocco and have shown the importance of some of its biological activity [20, 21]. However, there are no detailed studies on the action mechanisms of these activities, especially anticancer and anti-inflammation activities. Therefore, we investigated here the anti-inflammatory, antioxidant, antibacterial, and cytostatic effects on cancer cell lines of a propolis extract from the northern Morocco region. Dose-dependent cytostatic effects of propolis were evaluated in all three tested cancer cell lines. Propolis extracts also suppressed the TNF-*α* and IL-6 production in a dose-dependent manner in LPS-stimulated human peripheral blood mononuclear cells. Dose-dependent cytostatic effects of propolis were seen in all three cell lines. Propolis extracts suppressed the TNF-*α* and IL-6 production in a dose-dependent manner in LPS-stimulated PBMNCs, reaching control levels at 250 *μ*g/ml. Propolis increased the production of the anti-inflammatory IL-10 in a dose-dependent manner.

## 2. Materials and Methods

### 2.1. Chemicals, Reagents, and Equipment

#### 2.1.1. Chemicals and Reagents

2,2′-azino-bis (3-ethylbenzothiazoline-6-sulphonic acid) (ABTS), sulfuric acid, 2,2′-diphenyl-1-picrylhydrazyl (DPPH), potassium ferricyanide (K_3_[Fe(CN)_6_]), sodium carbonate, caffeic acid, *p*-coumaric acid, quercetin, gallic acid, cinnamic acid, naringenin, pinocembrin, galangin, rutin, pinobanksin, and chlorogenic acid were purchased from Sigma-Aldrich, Germany. Trichloroacetic acid, trisodium phosphate (Na_3_PO_4_), potassium dihydrogen phosphate (KH_2_PO_4_), and dipotassium hydrogen phosphate anhydrous (K_2_HPO_4_) were purchased from VWR, Leuven, Belgium, and chrysin was purchased from AbCam, UK. Ferulic acid was purchased from Acros Organics, USA. Iron (III) chloride was purchased from Buchs, Switzerland. Ammonium heptamolybdate ((NH_4_)_6_Mo_7_O_24_) was purchased from Pronalab, Lisbon, Portugal. Folin-Ciocalteu's phenol and AlCl3 were purchased from Panreac Quımica, Montcada i Reixac, Barcelona, Spain. Ascorbic acid, cell culture medium (DMEM and RPMI), 3-(4,5-dimethylthiazol-2-yl)-2,5-diphenyltetrazolium bromide (MTT), and triphenyltetrazolium chloride (TTC) were purchased from Sigma-Aldrich, USA. TNF-*α*, IL-6, and IL-10 kits were purchased from R&D Systems, Inc., USA. Mueller Hinton broth and Mueller Hinton agar were purchased from Biokar Diagnostics (Beauvais, France).

#### 2.1.2. Equipment

Specmate UV-VIS spectrophotometer (CLS-4048), pH meter (WTW Inolab pH 720), HPLC (Hitachi, Chrom-master; Japan), and microplate reader (Tecan Infinite M200; Tecan, Austria) were used.

### 2.2. Collection of Propolis Sample

Propolis was collected by scratching from the northern Morocco region (PNM); this region is known for olive trees in addition to other trees such as *Pinus*, *Quercus*, *Rosmarinus*, *Juniperus*, *Lavandula,* and *Pistacia*. The propolis sample was stored at room temperature (22–24°C) in airtight plastic containers until analysis.

### 2.3. Physicochemical and Antioxidant Content of Propolis Sample

In the present work, wax, balsam, and resin contents were measured in the PNM according to methods described by Papotti et al. [22]. The ash content in the sample was determined according to the method described by Imtara et al. [23]. The pH was measured according to the method described by Dias et al. [24]. The moisture content of samples was determined according to the method described by AOAC [25].

In order to determine the total antioxidant content, the total phenol, flavones, and flavonols contents were determined according to the method described by Imtara et al. [26]. The results of total phenol are expressed as the mg gallic acid/g of propolis, and for flavones and flavonols contents, the results are expressed as mg quercetin/g of propolis.

### 2.4. Antioxidant Activity of Propolis Sample

The total antioxidant capacity (TAA) was estimated by the phosphomolybdenum method according to the procedure described by Prieto et al. [27]. Total antioxidant capacity contents are expressed as mg of ascorbic acid equivalent per g of the propolis mass (mg AA/g).

The ability of sample for scavenging of free radical was determined by three methods: the scavenging activity of DPPH radical was measured according to the method described by Brand-Williams et al. [28], the scavenging activity ABTS radical was carried out according to the method of Miguel [29], and the reducing power was determined according to the method of Oyaizu [30]. The results of each test are expressed by IC50 value (concentration of samples is able to scavenge 50% of free radicals).

### 2.5. Determination of Phenolic Compounds by RP-HPLC Analysis

Hundred mg of PNM was extracted by sonication at 50°C using 70% ethanol (10 mL) for 30 min. The solution was cooled to room temperature and the volume was made up to 10 ml with 70% ethanol in volumetric flask, followed by centrifugation at 3500 rpm. The centrifuged supernatant was filtered through HPLC syringe filter (0.45 *μ*) before injection to HPLC. Pure compounds that are used as standards included caffeic acid, *p*-coumaric acid, ferulic acid, gallic acid, chlorogenic acid, rutin, quercetin, cinnamic acid, naringenin, pinocembrin, chrysin, galangin, and pinobanksin. Phenolic compounds of propolis were identified by comparing their retention times with those of pure standards. The results were obtained in mg/g of propolis.

### 2.6. Antibacterial Activity of Propolis Sample

The bacterial strains used in the present work were isolated at the University Hospital Hassan II and at the Microbiology Laboratory of the FMP, Fez. The *E. coli* BLSE (ATB: 87) BGN, *E. coli* (ATB: 57) B6N, *E. coli* (ATB: 97) BGM, and *Pseudomonas aeruginosa* strains are Gram-negative bacilli, and the *Streptococcus faecalis* and *Staphylococcus aureus* strains are Gram-positive cocci bacteria. The ability of propolis sample to inhibit bacterial growth was determined by qualitative and quantitative tests.

*The agar diffusion assay* was performed by Kirby-Bauer method [31]. With some modification, Mueller Hinton agar plates are inoculated by swabbing from the standardized suspensions (10^8^ cfu/mL). Then, Whatman paper disks (6 mm) are deposited on the surface of the preinoculated agar. Then, the disks are impregnated with 10 *μ*l of propolis extract. All plates were incubated at 37°C for 24 hours. After incubation, the diameters of the inhibition zones were measured.

*The minimum inhibitory concentration (MIC) and minimal bactericidal concentration (MBC) tests* were determined by microdilution method according to NCCLS standards in microplate (96-well plates) [32, 33]. With modification, ten concentrations of PNM were prepared in sterile tubes by successive dilutions 1/2 in hydroethanol (70%). A volume of 10 *μ*l of each concentration of this dilution series was mixed in microplate wells with 170 *μ*l of Mueller Hinton broth and 20 *μ*l of bacterial inoculums with a final microbial concentration 5 × 10^5^ CFU/ml. The final volume was 200 *μ*l and the concentration of ethanol in each well does not exceed 3.5%. The same percentage of ethanol was used as a negative control. After the plates are incubated at 37°C for 20 h, 40 *μ*l of triphenyltetrazolium chloride was added to each well. Then, the microplate was incubated for 2 hours. After incubation, the MIC is the lowest concentration that does not produce a red color [34]. To determine the MBCs, a portion from each well in which the concentration is ≥ MIC was subcultured on Muller–Hinton agar (MHA) and incubated at 37°C for 24 h. The MBC is defined as the lowest concentration of the extracts at which inoculated bacteria were 99.9% killed [35].

### 2.7. Cytotoxic and Cytostatic Effects of Propolis Sample

#### 2.7.1. Cell Culture

The human monocytic cell line THP-1 (ATCC 202-TIB) was purchased from American Type Culture Collection (Manassas, VA, USA). These cells are known to express various monocytes receptors and have been widely used as a model system for macrophage research [36, 37]. Cells were maintained in DMEM (Dulbecco's modified Eagle's medium) supplemented with 10% vol/vol inactivated fetal calf serum (FCS), 1% nonessential amino acids, 1% glutamine, 100 U/mL penicillin, and 10 *μ*g/ml streptomycin and kept in a humidified atmosphere of 5% CO_2_ at 37°C. Human colorectal carcinoma cell line HCT-116 (ATCC® CCL-247™) and breast cancer cell line MCF-7 (ATCC® HTB-22™) were grown in DMEM-5671 with a high glucose content (4.5 g/l), supplemented with 10% vol/vol inactivated fetal calf serum (FCS), 1% nonessential amino acids, 1% glutamine, 100 U/ml penicillin, and 10 *μ*g/ml streptomycin. The pH of the media for growing cells was maintained at 7.4 under 5% CO_2_ at 37°C.

#### 2.7.2. Cytotoxic and Cytostatic Effects in Monoculture System

For the cytotoxic and cytostatic assays, 20,000 cells/100 *μ*l and 5,000 cells/100 *μ*l media were seeded in 96-microtiter plates, respectively. Twenty-four hours later, cells were incubated with increasing concentrations of the PNM (0–1000 *μ*g/ml of culture media) for 24 hours and 72 hours for cytotoxic and cytostatic assays, respectively. Then, cell viability was measured using the MTT assay. Cell viability was defined as the ratio (expressed as a percentage) of absorbance of treated cells to untreated cells (control) [38].

#### 2.7.3. MTT Assay

MTT (tetrazolium dye) is widely used to measure the viability and/or the metabolic state of cultured cells [19]. Twenty-four hours after cell seeding, cells were treated with varying concentrations of PNM (0–1000 *μ*g/ml of culture media) for 24 hours at 37°C. Cells were then washed in phosphate buffered saline, incubated in serum-free RPMI to which MTT (500 *μ*g/mL) was added to each well (100 *μ*L), and incubated for a further four hours. After the removal of the medium, the cells were incubated for 15 minutes with 100 *μ*l of acidic isopropanol (0.08 N HCl) to dissolve the formazan crystals. The absorbance of the dissolved MTT formazan was measured at 570 nm in an ELISA reader. Viability was defined as the ratio (expressed as a percentage) of absorbance of propolis-treated cells to untreated control cells.

### 2.8. Anti-Inflammatory Activity of Propolis Sample

#### 2.8.1. Isolation of Peripheral Blood Mononuclear Cells (PBMNCs)

Blood samples were taken from 16 healthy volunteer students from the Arab America University (Jenin, Palestine), aged 19–21 years (7 males and 9 females).

Blood samples were withdrawn in heparin tube after filling consent and a written questionnaire. Venous blood (15 mL) was processed immediately after collection. Peripheral blood mononuclear cells (PBMNCs) were isolated using gradient centrifugation in Histopaque-1077 solution (Sigma-Aldrich) [37]. Separated PBMNCs were incubated in RPMI-1640 media supplemented with sodium bicarbonate, L-glutamine-penicillin-streptomycin solution (200 mM L-glutamine, 10,000 U penicillin, and 10 mg streptomycin/mL in 0.9% NaCl), and 10% vol/vol FCS. Isolated PBMNCs were seeded at a cell density of 1 × 10^6^ cells/ml in 24-well plates and exposed to propolis extract (125 *μ*g/mL and 250 *μ*g/ml) in a fresh serum-free medium in the absence and presence of *Escherichia coli* serotype O127 : B8 (5 *μ*g/mL). Cells were maintained at 37°C for 4, 6, and 20 hours at 5% CO_2_ and the levels of secreted IL-6, TNF-*α*, and IL-10 were determined as described below.

#### 2.8.2. Immunoassay for Cytokines

The anti-inflammatory activities were assessed by investigating propolis ability to alter the production of tumor necrosis factor-*α* (TNF-*α*) and the cytokines interleukin-6 (IL-6), and IL-10 in human peripheral blood mononuclear cells (PBMNCs) was costimulated with lipopolysaccharide (5 *μ*gLPS/mL). The amounts of secreted TNF-*α*, IL-6, and IL-10 were measured using a commercial ELISA kits (R&D Systems, Minneapolis, MN, USA). The absorbance at 450 nm was read by a microplate reader (model 680; Bio-Rad Laboratories, Mississauga, ON, Canada) with the wavelength correction being set at 550 nm. The amounts of TNF-*α*, IL-6, and IL-10 were calculated with the help of a standard curve, which was constructed using serial dilutions of cytokine standards provided with the kit.

## 3. Results and Discussion

### 3.1. Physicochemical Properties of Propolis Sample

Propolis is a resin that honeybees produce by mixing saliva and beeswax with exudates gathered from botanical sources. The amount of each of these compounds is often used as an indication of propolis quality, which depends on the phytogeographic and climatic conditions around the beehive [39, 40]. In the present work, the physicochemical properties of PNM showed that the concentrations of wax, resin, and balsam were 20.31 ± 1.03%, 59.01 ± 0.12%, and 16.4 ± 0.01%, respectively (Table 1). These values are within the range accepted for propolis by the Brazilian legislation [41] and were similar to those found in the Italian and Moroccan propolis [21, 22]. Moreover, high moisture in propolis is indicative of inadequate storage and manipulation conditions [42]. The moisture of the studied propolis sample was 1.01 ± 0.01%; this value is within the limit established by the Brazilian legislation (not more than 8%) [41]. The pH was somewhat acidic (5.1 ± 0.11), and it was similar to propolis pH found in other studies [43, 44].

With regard to the ash content, this analysis can identify a possible adulteration of the material through the presence of impurities [15]. The value of ash content in the PNM was 4.87 ± 0.01% which is lower than the upper limit which was defined as a quality standard in the propolis [41].

### 3.2. Bioactive Compounds and Pharmacological Activities of the Propolis Sample

The concentration and the type of bioactive compounds of propolis depend on many factors such as plant species, season of propolis harvesting, and geographical location of beehive collected [45].  Table 2 shows the values for the antioxidant content and antioxidant activity of the ethanol extract of PNM. The values of phenol and of flavone and flavonol were 141.46 ± 1.67 mg GAE/g, and 98.33 ± 1.19 mg QE/g, respectively. The total antioxidant capacity of the ethanol extract of PNM was 94.76 ± 1.91 mg AAE/g. These results are within the range reported in other studies [21, 46]. The capacity of the propolis samples to scavenge free radicals was evaluated and the results were expressed as IC50 mg/ml (Table 2). The results show a strong free radical scavenging with IC50 being 0.02 ± 0.002 for DPPH assay, 0.04 ± 0.001 for ABTS assay, and the same value for FRAP assay. The values of the IC50 are within the range reported in other propolis samples [46–48].

**Figure 1:**
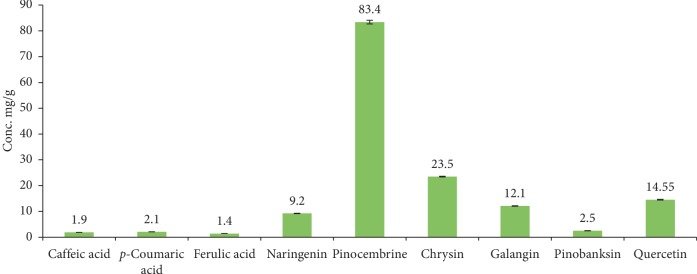
The concentration of phenolic compounds (mg/g) in PNM analyzed by HPLC, as described in Methods.

The pharmacologic properties of the phenolic compounds of propolis are documented in numerous scientific papers. These include, but are not limited to, anticancer, anti-inflammatory, antibacterial, and antioxidant activities [6, 49]. The PNM was analyzed via HPLC under the same chromatographic conditions for the determination of the phenolic compounds. The analysis identified nine phenolic compounds in propolis sample with different concentrations: caffeic acid, *p*-coumaric acid, ferulic acid, naringenin, pinocembrin, chrysin, galangin, pinobanksin, and quercetin (Figure 1). The major constituent in the sample was pinocembrin with concentration being 83.4 ± 0.71 mg/g of propolis. No detectable amounts of gallic acid, chlorogenic acid, and rutin were found in the propolis sample. The results of bioactive compounds identified in this article are in agreement with other studies, which have found that propolis samples collected in different countries contain many phenolic and flavonoid components at different concentrations such as caffeic acid, *p*-coumaric acid, ferulic acid, gallic acid, chlorogenic acid, rutin, quercetin, cinnamic acid, naringenin, pinocembrin, chrysin, CAPE, galangin, apigenin, kaempferol, chrysin, cinnamyl caffeate, and aromatic acids [50, 51]. Many studies documented the antimicrobial activity of these compounds against large number of bacteria [46, 52, 53].

**Figure 2:**
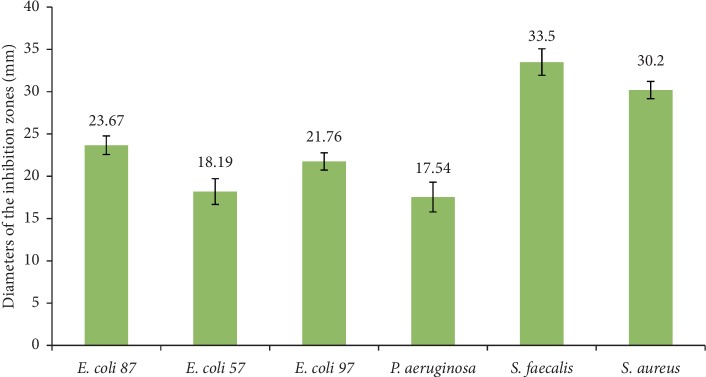
Antibacterial activity of PNM as detected by using the disc diffusion method. Whatman paper disks (6 mm) were impregnated with 10 *μ*l of propolis. The plates were incubated at 37°C for 24 hours subsequent to measuring the diameters of the inhibition zones in mm. The bacteria stains tested are indicated in the *x*-axis.

The diameters of bacterial growth inhibited by disc agar diffusion method of the propolis sample are shown in Figure 2. Comparing the six strains studied, the highest activity of PNM was observed on *S. faecalis* and *S. aureus* with zone inhibition values of 33.5 ± 1.57 and 30.2 ± 1.02 mm, respectively. The lowest activity was observed on *P. aeruginosa* with zone inhibition value of 17.54 ± 1.76 mm. The determination of the zone inhibitions of PNM on different bacteria strains showed that Gram-positive strains are more sensitive than Gram-negative bacteria. This is consistent with many studies [54, 55].

**Figure 3:**
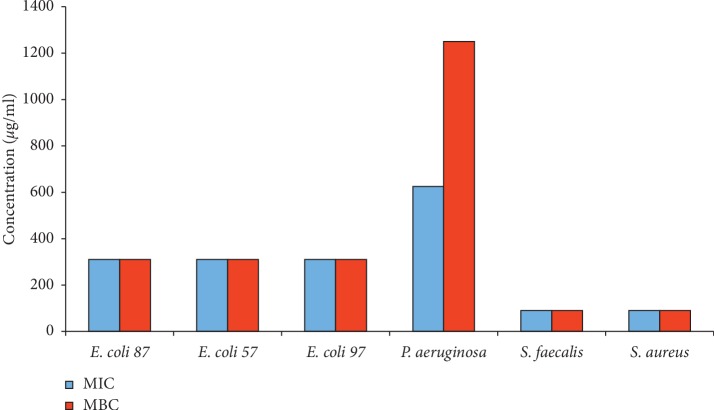
PNM minimum inhibitory concentration (MIC) and minimal bactericidal concentration (MBC) for six distinct bacteria strains as indicated in the *x*-axis.

In the present work, the minimum inhibitory concentration (MIC) is the lowest concentration of propolis, at which no bacterial growth was observed [46]. The MIC and MBC values for PNM on studied strains were 90–625 and 90–1250 *μ*g/mL, respectively (Figure 3). As found in the disc diffusion method, *S. faecalis* and *S. aureus* were the most sensitive and *P. aeruginosa* was the most resistant. The values of MICs reported in this study are lower than those reported in propolis from Canada [56], but similar to the results of MICs reported by other studies [46, 54].

### 3.3. Cytotoxic and Cytostatic Effects of Propolis in Monocultures of Cells from the Human THP-1, HCT-116, and MCF-7 Cell Lines

The research for new natural anticancer drugs is one of the main objectives of scientific research. As part of this work, we assessed here the capability of PNM to exert antiproliferative effects (cytostatic effects) in cancer cells at noncytotoxic concentrations.

**Figure 4:**
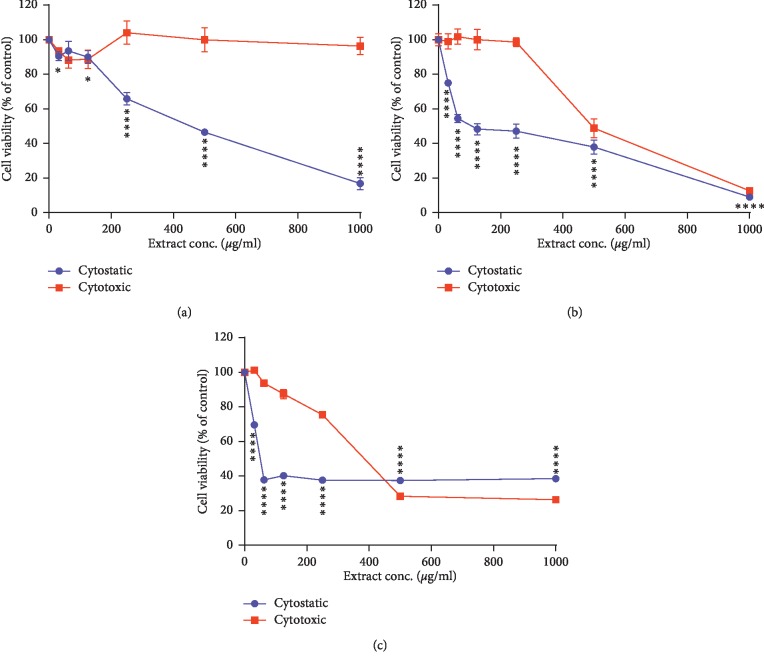
Cytostatic and cytotoxic activities of PNM in cells from the (a) HCT-116, (b) MCF-7, and (c) THP-1 cell lines. 20,000 cells/100 *μ*l and 5,000 cells/100 *μ*l media were seeded in 96-microtiter plates for the cytotoxic and cytostatic assays, respectively, and incubated with PEB (0–1000 *μ*g/ml of culture media) for 24 hours and 72 hours for cytotoxic and cytostatic assays, respectively. (^*∗*^)*p* < 0.05; (^*∗∗∗*^)*p* < 0.001.

No significant cytotoxic effects were observed in HCT cells at all PNM concentrations (up to 1000 *μ*g/ml) (Figure 4). Significant cytotoxic effects (LD50: lethal dose) were observed in MCF-7 cells and THP-1 at concentration higher than 494 and 385 *μ*g/ml, respectively (Table 3, Figure 4). Many reports suggest that cytotoxicity may largely vary in different samples of propolis. Szliszka et al. [57] reported that 50 g/mL ethanolic extract of propolis from southern Poland exhibited 25% cytotoxicity in prostate cancer cells. Seda Vatansever et al. [58] showed that ethanolic extract of propolis at a concentration of 125 g/mL is cytotoxic in MCF-7 cell line and reported differences in cytotoxic effects of seven different ethanolic extracts of propolis samples collected from the same location. Compared to these results, PNM exhibited relatively low grade of cytotoxic effects.

Dose-dependent cytostatic effects were measured in MCF-7 (IC50 of 108.9 *μ*g/ml), HCT (IC50 of 279.2 *μ*g/ml), and THP-1 (IC50 of 50.5 *μ*g/ml) cells with PNM concentrations higher than 30 *μ*g/ml and 100 *μ*g/ml, respectively [58]. Many reports have indicated that different types of honey and propolis extracts significantly inhibit cell growth and reduce the differentiation or proliferation of cells from various tumor cell lines [58]. For example, Imtara et al. characterized the phenolic compounds of twelve honey samples collected from different locations in Palestine and Morocco to evaluate their cytotoxic and cytostatic effects on cells from the human colorectal carcinoma cell line HCT-116 and breast cancer cell line MCF-7. They found a significant cytostatic effect after treatment of HCT cells as well as a strong correlation was observed between cytostatic activity of MCF cells and antioxidant content (phenols, flavonoids, and flavonol). Furthermore, a strong negative correlation was detected between the cytostatic activity in HCT cells and the contents of syringic acid and tannic acid. These results indicate that the traditionally known anticancer effects of honey might be mediated in part through cytostatic effects [38]. Another study investigated the anticancer effects of Indian ethanolic extract of propolis on four different cancer cell lines and demonstrated lower cytotoxicity effect of PNM evaluated by MTT assay (i.e., 250 g/mL) as compared to reported value [57, 58], which can be attributed to its different geographical origin. Apoptosis is an important phenomenon in chemotherapeutic agent which induced killing of cancer cells. Apoptosis induction is one of the mechanisms proposed for the therapeutic effects of propolis [59, 60]. Results obtained indicated that the mode of action of ethanolic extract of propolis is by inducing apoptosis, since DNA fragmentation is evidenced by the TUNEL assay. Seda Vatansever et al. have shown induction of caspases in MCF-7 cells [58]. Szliszka et al. discussed augmentation of TRAIL-induced apoptotic death in prostate cancer cells due to ethanolic extract of propolis [57]. The mechanisms by which our PNM exhibits its dose-dependent cytostatic effects need to be investigated.

### 3.4. Effect of Propolis Extracts on Proinflammatory Anti-Inflammatory Cytokines Production in PBMNCs

**Figure 5:**
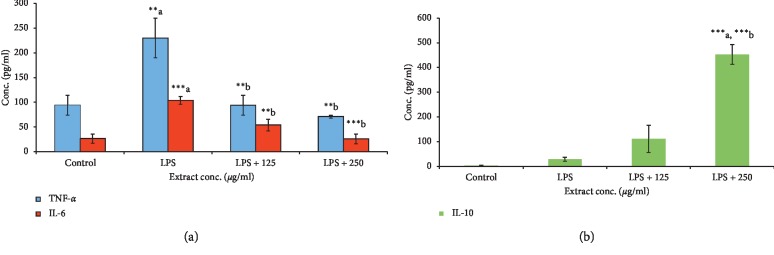
Dose-dependent inhibition of LPS-mediated production of TNF-*α*: IL-6 (a), and IL-10 (b) by PNM in PBMNCs. The bar heights represent the concentration means ± SD. ^a^Comparison between control group and all groups. ^b^Comparison between LPS group and all groups. ^*∗*^*p* < 0.05; ^*∗∗*^*p* < 0.01; ^*∗∗∗*^*p* < 0.001; ^*∗∗∗∗*^*p* < 0.0001.

The PBMC cellular model includes T and B cells (∼80%), natural killer cells (∼10%), and monocytes (∼10%). These blood cells play an important role in the adaptive immune response [61]. Several studies have found that propolis has immunological activities [62]. For example, Brazilian green propolis exhibited antioxidant and anti-inflammatory activities in LPS-stimulated macrophage cell line J774A.1 through the inhibition of the production of reactive oxygen species, nitric oxide, and proinflammatory cytokines (e.g., TNF-*α*, IL-1, and IL-6) [13, 63]. In the present study, PNM on its own showed no effect on the production levels of the proinflammatory cytokines TNF-*α* and IL-6 as well as the anti-inflammatory cytokine IL-10 in PBMNCs when compared to untreated control cells (data not shown). However, when PBMNCs were stimulated with LPS, the PNM significantly inhibited the secretion of TNF-*α* and IL-6 compared with LPS alone. Treatment with PNM completely inhibited the TNF-*α* and IL-6 secretion reaching control levels at extract concentration of 250 *μ*g/ml (Figure 5(a)). These results are in agreement with previous finding obtained in macrophage cell lines in which propolis compounds were found to have direct regulatory action on cytokine production. For example, neovestitol, an isoflavonoid from propolis, had an immune modulatory effect on LPS-stimulated cells from the macrophage cell line RAW264.7, where it clearly inhibited the production of nitric oxide (NO) proinflammatory cytokine. In Th1- and Th2-type T cells, propolis extracts and propolis active compounds caffeic acid, phenethyl ester, quercetin, and hesperidin strongly reduced the production of IL-1, IL-12, IL-2, and IL-4 and enhanced the production of transforming growth factor-*β*1 (TGF-*β*1) [13]. Zhang et al. reported that propolis-derived apigenin decreased the mRNA levels of IL-1, IL-6, and TNF-*α* in human THP-1-derived macrophages [16]. The levels of these proinflammatory cytokines were also significantly decreased by pinocembrin in macrophages (RAW264.7) [17]. In the same RAW264.7 line, the level of IL-6 and TNF-*α* cytokines was clearly reduced by propolis-derived galangin [18]. *In vivo* studies showed that propolis administration to C57BL/6 mice for 14 days led to the inhibition in the production of IL-1, IL-6, IL-2, IL-10, and IFN-*γ* by spleen cells [64]. In addition, ethanolic extract of Brazilian propolis reduced the expression of IL-17 in collagen-induced arthritis in mice [65].

IL-10 is a potent anti-inflammatory mediator; it reduces and terminates the ongoing inflammatory process [66, 67]. The effect of PNM on the secretion of the anti-inflammatory IL-10 cytokines by PBMNCs was measured by adding the extracts on their own and in combination with 10 *μ*g/mL LPS. LPS treatment of PBMNCs cells induced the secretion of a low level of IL-10 (30 ± 8 pg/ml). Compared to LPS-stimulated cells, treatment with propolis increased the secretion of IL-10 by 3.7-fold and 15-fold at propolis concentration of 125 *μ*g/mL and 250 *μ*g/mL (Figure 5(b)), respectively. Previous reports have clearly shown the antagonist effect of IL-10 on the secretion of proinflammatory cytokines [19, 68], suggesting that propolis-mediated inhibition of the LPS-induced secretion and mRNA expression of IL-6 and TNF-*α* may pass through the induction of IL-10 production. Several inflammatory diseases share the dual characteristic of a very low blood level of IL-10 and a high blood level of TNF-*α*. Furthermore, injection of the recombinant form of IL-10 decreased the blood concentrations of TNF-*α* that has proven beneficial for such diseases [19, 68, 69]. The ability of propolis extract to modulate both the proinflammatory and anti-inflammatory cytokines in LPS-activated PBMNCs represents an additional argument for the suggestion that it is an alternative or a complement that may help in the treatment and/or prevention of inflammatory diseases. Thus, our results support previous findings that suggest that propolis can be considered as potential natural anti-inflammatory agents that act by modulating the production of immune mediators.

## 4. Conclusion

Taken collectively, our results show that traditionally known anticancer effects of the PNM seem to be mediated in part through cytostatic effects and immunomodulatory effects. These include the inhibition of proinflammatory cytokines and stimulation of the anti-inflammatory cytokine in LPS-activated PBMNCs. The results of this study show that propolis from Morocco has important therapeutic activities especially in suppressing the TNF-*α* and IL-6 production and increasing the production of the anti-inflammatory IL-10. These multitarget actions including antibacterial, antioxidant, cytostatic, and immunomodulatory actions of the Moroccan propolis seem to be as a result of the presence of several active compounds which may act in a synergistic pathway. Our findings suggest the potential application of PNM in the pharmaceutical industry as well as in health foods and nutritional supplements.

## Figures and Tables

**Table 1:** Determination of physicochemical properties of PNM.

Sample	Wax (%)	Resin (%)	Balsam (%)	Moisture (%)	Ash (%)	pH
Moroccan propolis	20.31 ± 1.03	59.01 ± 0.12	1.11 ± 0.01	1.01 ± 0.01	4.87 ± 0.01	5.1 ± 0.11

**Table 2:** Determination of the content of total phenols, flavone, and flavonol and antioxidant activity by TAA, DPPH, ABTS, and FRAP of PNM.

Sample	Phenols (mg GAE/g)	Flavone and flavonol (mg QE/g)	TAA (mgAAE/g)	DPPH IC50 (mg/ml)	ABTS IC50 (mg/ml)	FRAP IC50 (mg/ml)
Moroccan propolis	141.46 ± 1.67	98.33 ± 1.19	94.76 ± 1.91	0.02 ± 0.002	0.04 ± 0.001	0.04 ± 0.001

**Table 3:** IC50 values (cytostatic) and DL50 values (cytotoxic) of the PNM were measured in three types of cells using the MTT test.

	HCT-116	MCF-7	THP-1
IC50 (*μ*g/ml)	479.22	108.88	50.54
LD 50 (*μ*g/ml)	—	493.97	385.11

## Data Availability

The data used to support the findings of this study are available from the corresponding author upon request.
